# Exosome biogenesis, secretion and function of exosomal miRNAs in skeletal muscle myogenesis

**DOI:** 10.1111/cpr.12857

**Published:** 2020-06-24

**Authors:** Binglin Yue, Haiyan Yang, Jian Wang, Wenxiu Ru, Jiyao Wu, Yongzheng Huang, Xianyong Lan, Chuzhao Lei, Hong Chen

**Affiliations:** ^1^ Key Laboratory of Animal Genetics, Breeding and Reproduction of Shaanxi Province College of Animal Science and Technology Northwest A&F University Yangling China

**Keywords:** exosomal miRNAs, exosome, non‐coding RNA, skeletal muscle

## Abstract

Exosomes are membrane‐bound extracellular vesicles that are produced in the endosomal compartment of most mammalian cell types and then released. Exosomes are effective carriers for the intercellular material transfer of material that can influence a series of physiological and pathological processes in recipient cells. Among loaded cargoes, non‐coding RNAs (ncRNAs) vary for the exosome‐producing cell and its homeostatic state, and characterization of the biogenesis and secretion of exosomal ncRNAs and the functions of these ncRNAs in skeletal muscle myogenesis remain preliminary. In this review, we will describe what is currently known of exosome biogenesis, release and uptake of exosomal ncRNAs, as well as the varied functions of exosomal miRNAs in skeletal muscle myogenesis.

## 1 BACKGROUND

As the largest organ of mammals, skeletal muscle is responsible for basic functions such as movement,^1^ respiration^2^ and metabolism^3^; however, studies of pathophysiological mechanism research have mostly focused on the activities of intracellular factors at the transcriptional,^4^ post‐transcriptional^5^ and translational levels.^6^ The formation of highly differentiated tissues and organs in multicellular organisms largely relies on a intricate network of cells with particular biological functions, where different cells within an organism establish efficient communication strategies to allow the exchange of biological information.^7^ A wide variety of cell types are present in skeletal muscle including stem cells, fibroblasts and immune cells, and fine‐tuned communication among these cells is vital to maintain homeostasis and function of skeletal muscle.^8^

**FIGURE 1:**
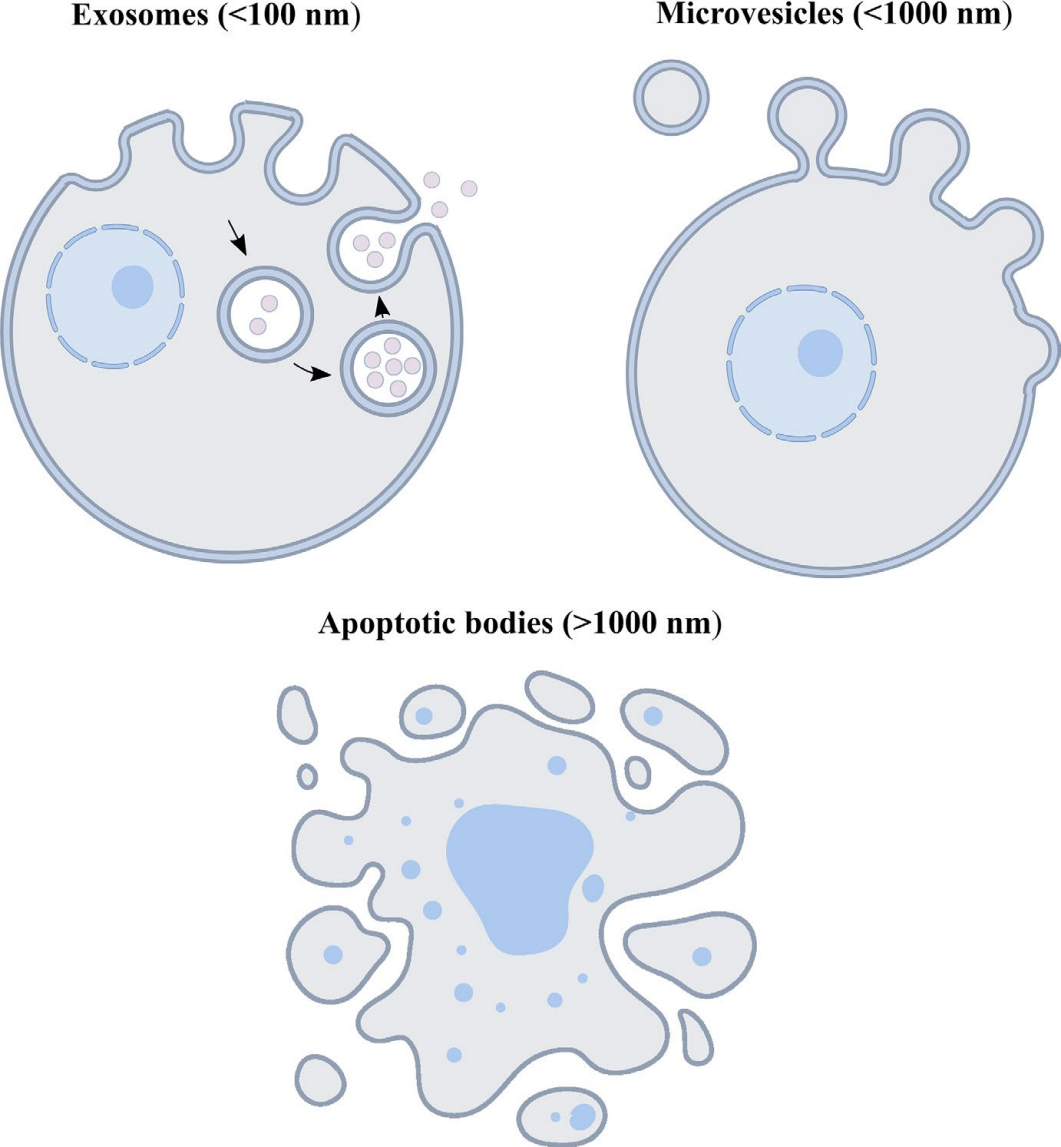
Schematic diagram of the biogenesis of exosomes, microvesicles and apoptotic bodies. Exosomes are endosome‐derived membrane vesicles, microvesicles are derived from budding of the plasma membrane, and apoptotic bodies arise from blebbing of apoptotic cell membrane

Cellular communication in mammals is generally mediated by tunnelling nanotubes or gap junctions,^9^ but recent studies of extracellular vesicles (EVs) derived from cells have expanded our understanding of cell‐to‐cell communication. EVs are natural carrier systems that can transfer nucleic acids, proteins, and lipids between donor and recipient cells in an autocrine, paracrine, and endocrine manner. EVs are generally divided into three main types according to their diameter, size and origin. Exosomes (<100 nm) are multivesicular body‐derived membrane vesicles, microvesicles (<1000 nm) form from budding of the plasma membrane, and apoptotic bodies (＞1000 nm) arise from blebbing of the apoptotic cell membrane^10^, ^11^ (Figure 1). Notably, exosomes can protect carried contents from being cleared by the mononuclear phagocyte system, making exosomes advantageous for cell communication.^12^

In recent years, the post‐transcriptional control exerted by non‐coding RNAs (ncRNAs) to regulate myogenesis has become better understood. Several ncRNAs, including long non‐coding RNAs (lncRNAs) and circRNAs, regulate target mRNAs expression by competitively binding miRNA response elements (MREs) with microRNAs (miRNAs).^13^, ^14^ There has been significant work done to characterize the potential roles of ncRNAs in exosomes, and some ncRNAs have been shown to be functional in recipient cells.^15^ This review will describe the origin and trafficking of exosomes and discuss the sorting mechanisms of exosomal ncRNAs and function of exosomal miRNAs in skeletal muscle myogenesis.

## 2 EXOSOME COMPOSITION

According to multi‐omics studies, exosomes contain different types of biomolecules including specific sets of proteins, lipids and nucleic acids.^16^, ^17^, ^18^ Because of the endosomal origin, exosomes are enriched in protein families associated with the formation of intraluminal vesicles (ILVs) (eg, tetraspanins, Tsg101 and Alix). Additionally, exosomes carry non‐specific proteins such as membrane fusion and transferring proteins (eg, annexins, Rab and flotillins), major histocompatibility complex (MHC) proteins (eg, MHC I and MHC II), heat shock proteins (eg, Hsc70 and Hsc90) and cytoskeleton proteins (eg, myosin, actin and tubulin).^19^ In addition to these common proteins, a wide range of cell type–specific proteins has been described in exosomes, which can vary dependent on pathophysiological conditions.^20^ In addition, exosomes are enriched in cholesterol, sphingomyelin, glycosphingolipids, phosphatidylserine and ceramide. These lipid contents are conserved and essential for maintenance of exosome morphology, exosome biogenesis, and regulation of homeostasis in recipient cells.^21^ Recently, exosome nucleic acids have been identified, including mRNAs and non‐coding RNAs such as miRNAs, lncRNAs, circRNAs, ribosomal RNAs (rRNAs), transfer RNAs (tRNAs), small nucleolar RNAs (snoRNAs), small nuclear RNAs (snRNAs) and piwi‐interacting RNAs (piRNAs). These RNAs are transferred from parent cells to recipient cells through exosomes and can exert special functional roles.^22^, ^23^ Exosome composition is highly heterogeneous depending on cellular origin and physiopathologic state, suggesting the recruitment of contents into exosome may be a regulated process.^24^

## 3 EXOSOME BIOGENESIS

**FIGURE 2:**
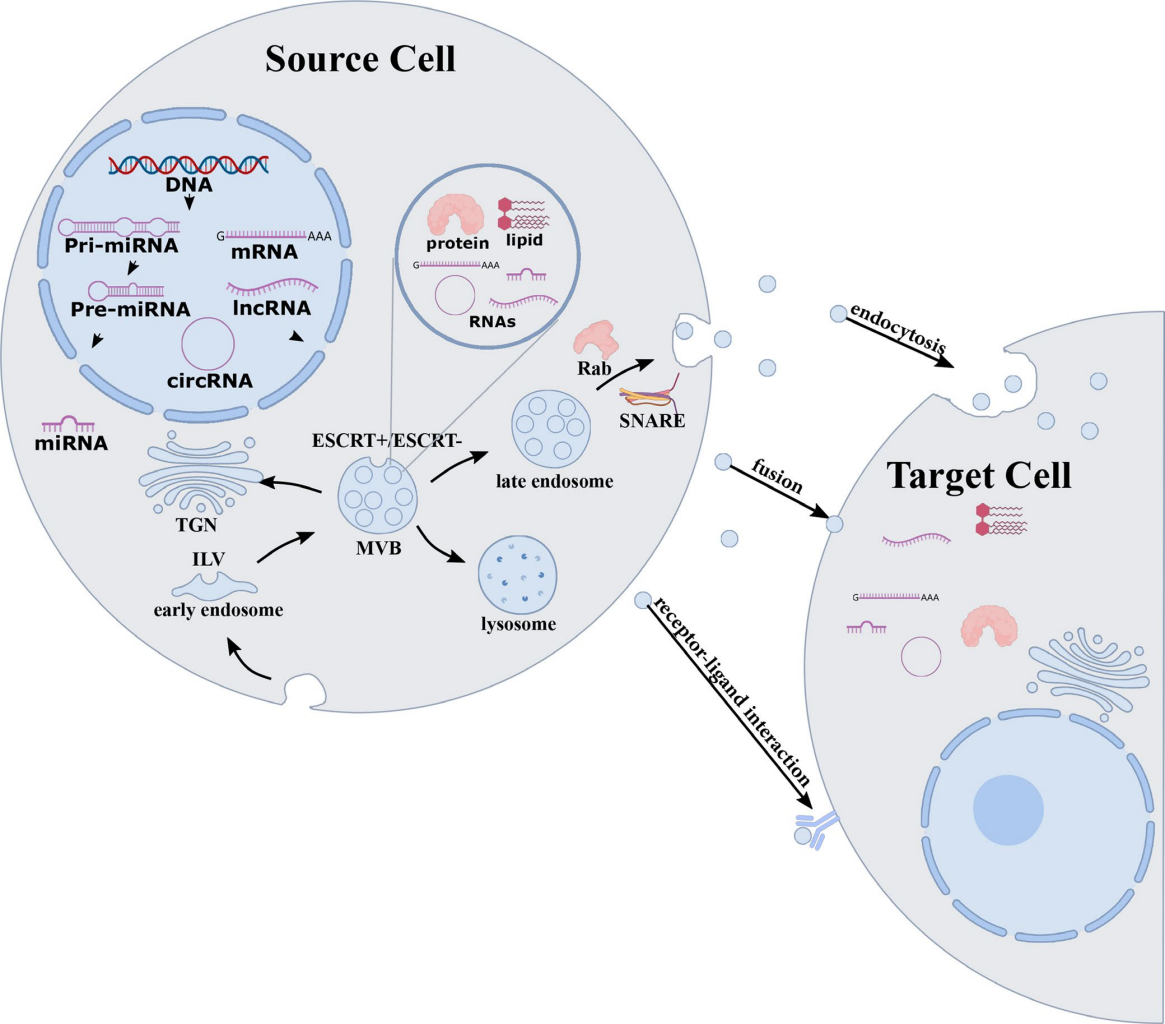
Schematic representation of the biogenesis, cargo and secretion of exosomes. Exosomes are formed by the invagination of the endocytic membrane and formation of ILV inside the cell. During maturation, the cargoes (RNAs, proteins and lipids) are incorporated into ILV through ESCRT‐dependent or ESCRT‐independent pathways, and the maturation of early endosomes gives rise to MVBs. MVBs can be transported to the trans‐Golgi network (TGN) for endosome recycling, delivered to lysosomes for degradation, or move along microtubules to fuse with the plasma membrane and release exosomes into the extracellular space. MVB fusion with the cellular membrane is a fine‐tuned process, which requires several crucial factors such as Rab GTPases and SNARE complexes. Exosomal cargoes from the source cell can be further delivered to target cells via endocytosis, direct membrane fusion and receptor‐ligand interaction

Exosomes originate with inward budding of the plasma membrane to form early endosomes, whose membranes then partly invaginate and bud into surrounding lumina with cytoplasmic content to form ILVs.^25^ Late endosomal structures containing dozens of ILVs are known as multivesicular bodies (MVBs), which are eventually transported to the trans‐Golgi network (TGN) for endosome recycling, delivered to lysosomes for degradation of all carried material, or fuse with the plasma membrane and release exosomes into the extracellular space^26^ (Figure 2). Exosome biogenesis and secretion require formation of an endosomal‐sorting complex that is required for transport (ESCRT), as reviewed recently.^27^ ESCRT is comprised of four complexes (ESCRT‐0, ESCRT‐I, ESCRT‐II and ESCRT‐III) and associated proteins (VPS4, Tsg101 and ALIX). ESCRT‐0 sorts ubiquitinated cargo proteins into the lipid domain, ESCRT‐I and ESCRT‐II induce membrane deformation to form the stable membrane neck, and recruitment of the Vps4 complex to ESCRT‐III drives vesicle neck scission and the dissociation and recycling of the ESCRT‐III complex.^28^

A wide array of studies suggested an ESCRT‐independent pathway in exosome biogenesis and cargo loading, which involves lipids and associated protein such as tetraspanin.^29^ In contrast to proteins sorted by ESCRT, RNA loading into exosomes appears lipid‐mediated, in a process that is dependent on self‐organizing lipid and cargo domains. Specific nucleotide sequences exhibit enhanced affinity for the phospholipid bilayer, which depends on factors such as lipid structure (lipid rafts), hydrophobic modifications and sphingosine at a physiological concentration in rafted membranes.^30^ Lipid rafts are subdomains of the plasma membrane that are enriched in cholesterol, sphingolipids and glycosyl‐phosphatidylinositol (GPI)‐anchored proteins, whose association with proteins or molecules might facilitate their secretion through exosomes.^31^ The presence of ceramide, lyso‐phospholipid and glycosphingolipid molecules on limiting membrane induces the spontaneous budding‐in process to produce ILVs.^32^ The ceramide converts to sphingosine and sphingosin1‐phosphate (S1P) in the presence of ceramidase and sphingosine kinase, and the continuous activation of sphingosine 1‐phosphate receptors on limiting membrane mediates tetraspanin sorting into ILVs.^33^, ^34^ Tetraspanin is a protein superfamily of cell surface‐associated membrane proteins characterized by four transmembrane domains. Tetraspanins organize membrane microdomains called tetraspanin‐enriched microdomains (TEMs) with a wide diversity of transmembrane and cytosolic signalling proteins.^35^ As the first characterized tetraspanin, CD63 functions in ESCRT‐independent ILV formation.^36^ Interestingly, a study in mammalian cells found that the absence of the ESCRT machinery did not block the formation of MVB vesicles, but resulted in impaired cargo‐sorting into ILVs and variations in ILV number and size, suggesting exosome biogenesis might be a coordinated process involving ESCRT‐dependent and ESCRT‐independent pathways.^37^

## 4 EXOSOME RELEASE AND UPTAKE

Exosome release depends on transport and plasma membrane fusion of the secretory MVBs after inward budding of ILVs, which requires several crucial factors including molecular switches (small GTPase), cytoskeleton (microtubule and microfilament), molecular motors (dynein and kinesin) and the membrane fusion apparatus (SNARE complex).^38^ Rab GTPase is the most important factor, with more than 70 subtypes located on the surface of membranes, where they can regulate vesicle traffic including budding, motility and fusion.^39^, ^40^ For instance, Rab27 binds to its corresponding effector proteins (Slp4, Slac2‐b and Munc13‐4) to regulate the transport and fusion of secretory vesicles,^41^ and Rab35 localizes to the surface of oligodendroglia cells in a GTP‐dependent manner, controlling the docking of endocytic vesicles with the plasma membrane.^42^ During secretory MVB transport, MVBs not only move along the microtubule cytoskeleton, but also require the action of molecular motors in plasma membrane directional transport. The microtubule cytoskeleton and molecular motors exhibit significant polarity distribution inside the cells, allowing variation in the distribution of MVBs. MVBs and plasma membranes can fuse via mediation by Rab and the corresponding effector on the MVB membrane.^43^, ^44^, ^45^ The details of the fusion process remain elusive, but a protein family collectively known as soluble N‐ethylmaleimide‐sensitive factor attachment protein receptors (SNAREs) is widely accepted as the core machine for membrane fusion. The specific pairing of vesicle SNAREs (v‐SNAREs) with the cognate target membrane SNAREs (t‐SNAREs) forms the SNARE complex, which drives fusion of two opposing membranes in a zipper‐like fashion. Accordingly, MVBs fuse with the plasma membrane and release exosomes into the extracellular space.^46^, ^47^

After secretion, signals in exosomes can be transferred to recipient cells through at least three different mechanisms: endocytosis, direct membrane fusion and receptor‐ligand interactions. Recent research increasingly suggests that endocytosis is the primary method for uptake of exosomes. Exosomes can be internalized by clathrin‐, caveolin‐ and lipid raft‐mediated endocytosis for different specific recipient cell types.^48^ When endocytosed, exosomes may subsequently merge into endosomes or be moved to lysosomes for degradation.^49^ In addition, the exosomal membrane can fuse with the plasma membrane to deliver contents into recipient cells, or bind to cognate receptors on the recipient cell membrane, subsequently eliciting intracellular signalling cascades^50^, ^51^ (Figure 2).

## 5 EXOSOMAL RNAS

The presence of exosomal mRNAs and miRNAs was first reported in murine and human mast cell lines (MC/9 and HMC‐1, respectively) in 2007; subsequently, other types of RNA have been confirmed in exosomes.^52^ Some exosomal RNAs are tissue specific, and others are present in exosomes regardless of cellular origin, suggesting potential different mechanisms related to the sorting of RNA cargoes.^53^ Thanks to technical advances in the separation and detection of exosomal RNAs, growing evidence shows that exosome‐mediated transfer of RNAs between cells is possible and functional. Here, we focus on mRNAs, miRNAs, lncRNAs and circRNAs identified in exosomes.

### 5.1 Exosomal mRNAs

Exosomal mRNAs have been identified as important exosomal cargos and functional regulators of cell behaviour under different physiological and pathological conditions. Previous studies showed that mRNA patterns in exosomes are substantially different from those in their donor cells, and can vary with cell types and species. For example, microarray analysis by Valadi et al identified approximately 1300 mRNAs from MC/9 exosomes, 270 of which were not detectable in the donor cell. Other microarray assessments of mRNA populations in EVs and their donor glioblastoma cells found that approximately 4700 mRNAs were detected exclusively in EVs. MC/9 exosomal mRNAs have been linked to ontologies such as cellular development, protein synthesis and RNA post‐transcriptional modification, whereas specific mRNAs detected in glioblastoma‐derived exosomes have been associated with cell proliferation, cell migration and immune response.^52^, ^54^ There may be a dedicated mechanism for selective targeting of mRNAs into exosomes, and motifs enriched in mRNA with a 3′‐UTR may potentially serve as cis‐acting elements targeting mRNAs to exosomes.^55^ Interestingly, after transfer of murine exosomal RNA to the HMC‐1, new murine proteins were observed in the HMC‐1, indicating that exosomal mRNAs can be translated in recipient cells.^52^ A later report suggested that TGF‐β1 mRNA transported by injured tubular cell exosomes can initiate repair or regenerative responses in fibroblasts.^56^ In addition, serum exosomes were discovered to contain full‐length ECRG4 mRNA, whose internalization into TCA8113 cells led to suppressor phenotype in inflammation, angiogenesis and cell proliferation of recipient cells.^57^ Most previous studies only demonstrated EV‐derived mRNA transfer between cells in vitro, and it was unclear whether biologically shuttling of mRNA can actually occur in vivo. To investigate further, the CRE recombinase system was applied in the absence of any ex vivo manipulation in mouse, and the results showed that CRE mRNA secreted by immune cell EVs was transmitted to recipient non‐immune cells and subsequently translated into functional CRE protein.^58^ In summary, many exosomal mRNAs can be transferred to recipient cells, where they are then translated and can contribute to the protein expression programs of recipient cells.

### 5.2 Exosomal miRNAs

Among exosomal cargo biomolecules, miRNAs have attracted the most attention, due to their high conservation across species and extensive regulatory roles in gene expression.^59^ Similar to protein‐coding genes, most miRNA genes are transcribed by RNA polymerase II to form primary transcripts (pri‐miRNAs) that are further processed by the RNase III DROSHA and DICER1 into mature ~22‐nt miRNA duplexes.^60^ Most single‐stranded miRNAs negatively regulate gene expression by targeting the 3′‐UTR of target genes, under the control of RNA‐induced silencing complex (RISC complex) to regulate biological homeostasis and pathological processes at the intracellular level.^61^ The discovery of miRNAs in exosomes suggests that miRNAs can be directly delivered to target cells via exosomes, resulting in the functional modulation of mRNA targets. For instance, vascular smooth muscle cells (VSMCs)‐derived exosomes mediate the transfer of KLF5‐induced miR‐155 from smooth muscle cells (SMCs) to endothelial cells (ECs), which induces endothelial injury and promotes atherosclerosis by repressing zonula occludens‐1 (ZO‐1).^62^ Exosomal miR‐21 secreted by cardiac progenitor cells (CPC) under oxidative stress inhibits the apoptosis of H9C2 cardiac cells by targeting PDCD4.^63^ Similarly, mouse brain endothelial cell (mBEC) exosomes were suggested to transmit EC‐specific miR‐126 to mouse cardiomyocytes, and stroke‐induced decrease of miR‐126 may subsequently induce cardiac hypertrophy by increasing the levels of MCP1 and VCAM1.^64^ Recent studies also demonstrate that adipocyte‐derived exosomal miR‐27a and adipose tissue macrophages (ATMs)‐derived miR‐155 all result in insulin resistance in myocytes by targeting PPARγ.^65^, ^66^ In addition to their roles to regulate gene expression after exosomal transfer, miRNAs such as miR‐21, miR‐29a and let‐7b may also activate Toll‐like receptors (TLRs) and induce cell activation and cytokine production under certain circumstances.^67^, ^68^

There is growing evidence that the miRNA profiles of exosomes differ from that of the parent cells, and exosomal miRNA expression levels are altered under different pathophysiological conditions, indicating active sorting of miRNAs into exosomes. Export of miRNA into exosomes occurs via an ESCRT‐independent pathway. The neural sphingomyelinase 2 (nSMase2)‐dependent pathway was first found to guide packaging of miRNAs into exosomes. As a rate‐limiting enzyme of ceramide biosynthesis, expression of nSMase2 is linearly correlated with the levels of exosomal miRNAs.^69^ Current studies have suggested roles for RNA‐binding proteins in miRNA sorting, for example, Ago2 levels and phosphorylation control secretion of some miRNAs in exosomes.^70^ Additionally, heterogeneous nuclear ribonucleoproteins (hnRNP) family proteins can bind to exosomal miRNAs and induce the loading of miRNAs into exosomes, and hnRNPA2B1 and hnRNPA1 recognize specific miRNA tetranucleotide sequences and load them into exosomes.^71^ In another example, the RNA‐binding protein Y‐box protein I (YBX1) controls the secretion of miR‐223 in exosomes in HEK293T cells.^72^ A previous finding in B cells suggested that post‐transcriptional modifications, notably 3′ end uridylation, may also contribute to direct miRNA sorting into EVs.^73^ Furthermore, both cellular levels of miRNAs or their target mRNAs can determine exosomal miRNA sorting in MVBs.^74^ Collectively, these observations suggest there are intricate mechanisms linking miRNA availability with exosome loading; however, the efficiency and regulation of miRNA sorting and the activity of exosomal miRNA in recipient cells remain unclear.

### 5.3 Exosomal lncRNAs and circRNAs

Deep sequencing of exosomal RNA derived from different cell types reveals many lncRNAs and circRNAs, which exhibit transcript sponge functions.^75^, ^76^ LncRNAs are defined as mRNA‐like transcripts longer than 200 nucleotides with limited protein‐coding capacity. Many lncRNAs have been found to be involved in a variety of cellular processes, ranging from chromatin organization, gene transcription and gene post‐transcriptional regulation to protein translation.^77^ Numerous lncRNAs have been detected in exosomes from bodily fluids and tumour cells, and these lncRNAs act in cell‐to‐cell communication during cancer pathogenesis. For example, lncARSR promotes sunitinib resistance via competitively binding miR‐34/miR‐449 to facilitate AXL and c‐MET expression in renal cell carcinoma (RCC) cells, and intercellular transfer of lncARSR by exosomes disseminated sunitinib resistance. The packaging of lncARSR into exosomes may be mediated by hnRNPA2B1, which specifically binds to the sequence at the 5′ end of lncARSR.^78^ As an lncRNA that is abundantly expressed in a majority of human cancers, H19 can be transferred from carcinoma‐associated fibroblasts (CAFs) to colorectal cancer cells (CRCs) through exosomes, where it can activate the β‐catenin pathway as a competing endogenous RNA sponge for miR‐141 in CRCs, promoting the stemness and chemoresistance of CRCs.^75^ Similarly, both exosomal and hepatic H19 levels are positively correlated with hepatic fibrosis in biliary atresia (BA) patients, and cholangiocyte‐derived exosomes delivered H19 to hepatocytes directly or via circulation and eventually promoted cholestatic liver injury.^79^ Although the exact regulatory mechanisms are not yet fully elucidated, specific proteins termed fundamental lncRNAs carriers can control lncRNA sorting into exosomes, and specific lncRNA–RBP complexes might capture specific microRNAs and sort them into exosomes.^80^

Circular RNAs, ncRNAs characterized by covalently closed‐loop structures without 5′ caps and 3′ poly‐tails, giving circRNAs greater stability than other RNAs in eukaryotes, act in various physiopathological processes.^81^ Similar to exosomal lncRNAs, circRNAs can be transferred through exosomes between donor cells and recipient cells, and typically act as ceRNA to modulate the tumour microenvironment. Exosomal circ‐IARS secreted by pancreatic cancer cells entered human microvascular vein endothelial cells (HUVECs) and then competitively adsorbed miR‐122 to inhibit target gene RhoA activity, which then altered the expression of F‐actin and ZO‐1 to enhance endothelial monolayer permeability and promote tumour metastasis.^82^ The ciRS‐133 is up‐regulated in gastric cancer (GC), enters pre‐adipocytes through exosomes derived from GC cells and then promotes the differentiation of pre‐adipocytes into brown‐like cells by targeting the miR‐133/PRDM16 axis.^83^ Exosomal circPTGR1 derived from LM3 cells (a high‐metastatic hepatocellular cell line) similarly enhanced the metastatic and invasive abilities of 97 L (a low‐metastatic hepatocellular cell line) and HepG2 cells (a non‐metastatic hepatocellular cell line) by affecting the miR449a–MET pathway of the recipient cells.^84^ Expression levels of some circRNAs are enriched in EV relative to the levels of their linear counterparts compared to the ratio in donor cells, and exosomes may represent a mechanism for intracellular circRNAs clearance, contributing to cell‐to‐cell communication.^85^, ^86^

## 6 EXOSOMES AND MYOGENESIS

Vertebrate skeletal muscle is mostly derived from somites of the paraxial mesoderm, and cells undergo complex cycles of hyperplasia and hypertrophy.^87^, ^88^ Myogenic cells proliferate to multiply cell numbers and then fuse to form multinucleated myotubes, which can then undergo further differentiation during embryogenesis, post‐natal growth and regeneration.^89^ In general, myogenesis is controlled by a well‐established transcriptional hierarchy including myogenic regulatory factors (MRFs) and members of the myocyte enhancer factor 2 (MEF2) family, which precisely coordinates the activities of a set of muscle genes.^90^ Skeletal muscle has been widely considered to be a secretory organ that can release factors into circulation in response to numerous environmental and physiological challenges.^91^ These factors secreted from skeletal muscle are termed myokines, and these factors may influence myogenesis or further modulate the homeostasis of peripheral organs such as adipose tissue, liver and bone.^92^ In contrast, skeletal myogenesis also requires coordinated action of various intercellular signalling factors, including proteins, lipids and nucleic acids.^93^ Skeletal myogenesis not only relies on coordinated intracellular events, but also involves coordinated intercellular interactions.

Recently, exosomes have been increasingly recognized as excellent transmitters that deliver intercellular signals between myocyte and other cells, resulting in phenotypic changes in target cells.^94^ Guescini et al demonstrated that cultured C2C12 cells could release exosomes that carry mtDNA and some functionally relevant proteins. The authors surmised that mtDNA can be imported into the mitochondria of target cells through the exosomal route, and C2C12 exosomes can deliver signal transduction machinery to target cells.^95^ Subsequent studies have provided evidence for the function of skeletal muscle‐secreted exosomes. High‐fat‐diet feeding in mice induces the release of exosomes by skeletal muscles, which subsequently induces myoblast proliferation and altered the expression of genes involved in the regulation of muscle cell cycle and differentiation in vitro.^96^ Moreover, lipidomic analyses here indicated exosomes likely transfer lipids between muscle cells, suggesting that exosomes act as “paracrine‐like” signals to modify muscle homeostasis. A previous study showed C2C12 and human myoblasts cultured in exosome‐depleted serum formed fewer myotubes than myoblasts grown in normal serum with increased level of myostatin and reduced myog expression; however, this condition was not reversed when the medium was switched to classical cell media. Additionally, myomiRs such as miR‐1, miR‐206 and miR‐133a are significantly decreased during myoblast proliferation. These result suggest that skeletal muscle‐secreted exosomes may contain specific biochemical signals for skeletal muscle myogenesis, implicating exosomes in crosstalk between mature muscle and myoblasts.^97^ To investigate the roles of muscle‐derived exosomes in intercellular communications, proteomic and transcriptomic levels of exosomes released from skeletal muscles were characterized. Proteomic analysis of exosomes secreted from C2C12 myoblasts and myotubes revealed that muscle release different populations of exosomes, and protein compositions may change during development. Myotube‐secreted exosomes reduced myoblast proliferation and induced cells to differentiate, supporting the hypothesis that muscle‐derived exosomes could participate in the dialog between myoblasts and myotubes.^98^ In a different experiment, miRNA repertoires of exosomes released from C2C12 myoblasts and myotubes revealed distinct specific subsets of miRNAs, and myotube‐secreted exosomes miRNAs targeted *Sirt1* in myoblasts to regulate differentiation.^99^ Exosomes from differentiating human skeletal muscle cells (HSkMs) are rich in various myogenic factors such as tumour necrosis factor (TNF), insulin‐like growth factors (IGFs) and fibroblast growth factor‐2 (FGF2). Differentiating HSkM exosomes significantly induce the myogenic differentiation of human adipose‐derived stem cells (HASCs), with increased fusion index and increased expression of myogenic genes (*ACTA1, MYOD1, DAG1*, *DES*, *TNNT1* and *MYH1/2*) in HASCs. There may also be paracrine effects, and differentiating HSkM exosomes can carry diverse myogenic factors to trigger myogenic signalling pathways inside target cells, resulting in skeletal myogenesis.^100^ In addition, exposure to C2C12 myotube‐derived exosomes facilitates cell proliferation, migration and tube formation in HUVECs. Consistently, nuclear factor‐κB signalling was elevated in response to exosomal stimulation, and miR‐130a was particularly enriched in C2C12 myotube‐derived exosomes and successfully transferred into HUVECs.^101^ Moreover, differentiating C2C12 exosomes treatment increased motor neuron survival and neurite outgrowth of NSC‐34 motor neuron cell line with dose dependence, demonstrating paracrine potential for muscle‐specific exosomes.^102^ These lines of evidence suggest that skeletal muscle‐secreted exosomes significantly mediate cell‐to‐cell communication inside or outside the skeletal muscle tissue. Because exosomes carry numerous proteic, lipidic and nucleic acid components, they can affect multiple signalling pathways in target cells.

**FIGURE 3:**
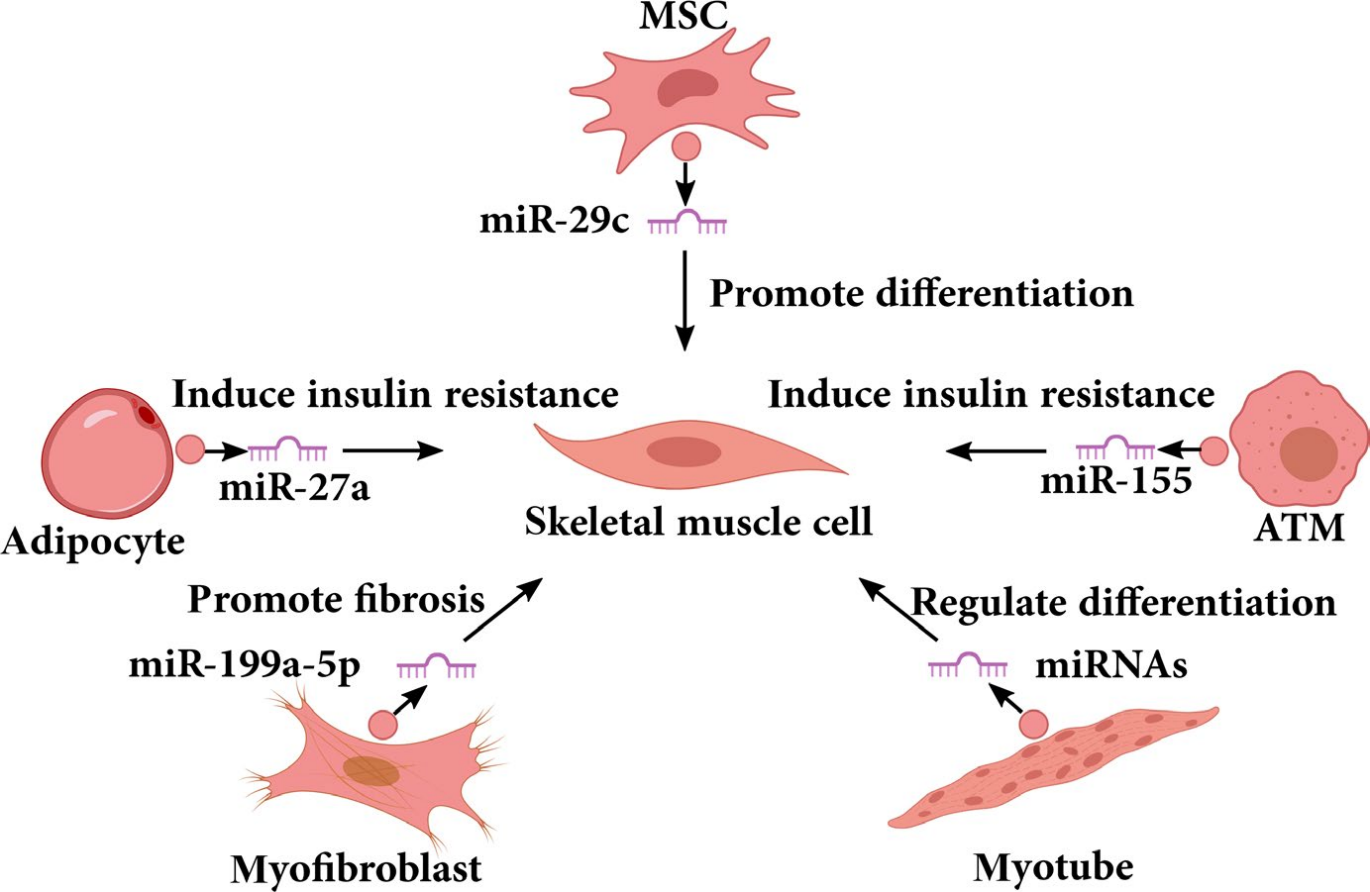
Examples of exosomal miRNAs that play a role in skeletal muscle myogenesis. A series of functional studies show that exosomes derived from different cells regulate skeletal muscle myogenesis by transferring miRNAs. MSC, mesenchymal stromal cells; ATM, adipose tissue macrophages

Using fluorescent labelling approaches, muscle cells were discovered to not only secrete exosomes, but also able to easily endocytose exosomes (Figure 3). Mesenchymal stromal cells (MSCs) are mesoderm‐derived multipotent stem cells that can differentiate into adipogenic, myogenic and osteogenic lineages.^103^ The cellular effects of MSCs are mainly attributed to paracrine effects mediated by a variety of chemokines, cytokines or growth factors. According to a recent study, human bone‐marrow MSC‐derived exosomes exert a novel paracrine effect on skeletal muscle by promoting both the proliferation and differentiation of C2C12 cells, which is at least partly mediated by miR‐494.^104^ Another study demonstrated that MSCs derived from placenta can release exosomes with high expression of miR‐29, and exosome treatment increased the differentiation of human muscle cells by transferring exosomal miR‐29c.^105^ Variation in specific miRNAs in exosomes may be related to the source of MSCs and the type of target myoblasts.

Adipose tissue has been suggested to be a major source of circulating exosomal miRNAs in obese states, and adipocyte‐derived exosomal miR‐27a was confirmed to result in insulin resistance in skeletal muscle by targeting PPARγ.^65^ The miR‐27a is highly expressed in adipose tissue and represses adipogenesis by directly targeting PPARγ. Activated PPARγ maintains glucose homeostasis in skeletal muscle by the modulation of genes harbouring PPAR response elements (PPREs).^106^, ^107^ Enriched miR‐199a‐5p was observed in exosomes released by myofibroblasts of a Duchenne muscular dystrophy (DMD) patient, and further transferred to reduce its target *caveolin‐1* expression in normal skeletal muscle, consequently promoting skeletal muscle fibrosis.^108^ Despite cumulating evidence for the biological function of exosomes, the biogenesis, secretion and molecular function of exosomes remain unclear in skeletal muscle myogenesis. Most studies of myogenesis‐related exosomes have focused on exosomal miRNAs, with only limited identification and determination of functional roles of other exosomal RNAs.

## 7 CONCLUSIONS AND PERSPECTIVES

As nanoscale biological vesicles, exosomes protect contents from degradation and facilitate their intercellular transmission, with significant impact on cell physiopathological processes such as immune defence, cell proliferation and tumour metastasis. Here, we described the mechanisms of exosome biogenesis, release, uptake, the potential sorting mechanism and what is known of the function of exosomal miRNAs in skeletal muscle myogenesis. No universal mechanism of exosome formation has emerged, and it remains controversial whether the differences between multiple hypotheses described above are due to differences in the exosome formation mechanism of different cells, or to differences in the nature of the vesicles. With current technical limitations, it is difficult to isolate pure exosomes and specific subtypes of exosomes from mixtures of different vesicle types, limiting exploration of function and mechanism.^48^ Different separation methods to isolate exosomes from the same cell type result in different proteomic characteristics, further complicating the situation.^109^ In addition, the complex diversity of EVs needs to be acknowledged: different vesicle types may carry different specific cargoes, use different cellular uptake routes and employ a different uptake ratio.^93^ Therefore, the purification of exosomes is required for more careful investigation.

In‐depth understanding of exosomal ncRNAs will better clarify cell‐to‐cell interactions. Our review highlights the importance of ncRNA as extracellular signalling molecules: ribonucleoprotein (RNP) complexes implicate in exosomal RNAs sorting and uptake, and exosomal ncRNAs mainly function as sponges for miRNA to affect the cellular processes of receptor cells. However, our current understanding of exosomal RNAs is incomplete. For instance, it is uncertain whether exosomal RNAs sorting and uptake is an active or a passive process. Current studies on exosomal RNAs functions have generally been performed with a large excess of exosomes, and it is unclear whether endogenous exosomes are feasible as functional RNAs transfer carriers in native physiological settings. Additionally, the content of exosomes depends on cell type from which they originate and biological context, and if RNA molecules in exosomes are intact or partially degraded is unclear, but may affect exosomal RNA functions in recipient cells.

Given the importance of skeletal muscle in movement, respiration and metabolism, we discussed the significant biological activity of muscle‐related exosomes mediated mainly by their cargo of miRNAs, suggesting the secretion and uptake of exosomal miRNAs by muscle cells is a key mode of communication to regulate skeletal muscle myogenesis. During evolution, eukaryotes developed elegant cell‐to‐cell strategies to adapt to their surrounding environment. Tissues do not function in isolation from one another, and factors secreted by special cells can subsequently act on a cell directly (autocrine signalling) or interact with neighbouring (paracrine signalling) and distant (endocrine signalling) cells. Exosome‐secreted‐myomiRs (ex‐miRNAs) may act as “myokine” signals to transfer gene regulatory information in the muscle niche, or other exosomal RNAs may be important. With better understanding of the RNAs that exosomes carry, future research should aim to determine the factors that govern release and uptake of exosomes and the mechanisms of crosstalk in skeletal muscle myogenesis. Furthermore, the complexity of skeletal muscle composition needs to be acknowledged in muscle physiology research. Due to the difficulty in obtaining exosomes from tissues, most work to characterize muscle exosomes has been conducted using the C2C12 cell line.

In conclusion, despite growing evidence for a role in communication under pathological conditions, our current knowledge of exosome physiology, diversity, internalization and cargo delivery is still limited, and how exosomes shape skeletal muscle myogenesis remains unknown. Exosomes may be associated with RNA delivery, and greater understanding of the muscle exosome field requires research in cellular functional phenotype and exosomal RNA sorting mechanisms.

## CONFLICT OF INTEREST

The authors declare no competing interests.

## AUTHOR CONTRIBUTIONS

Binglin Yue and Hong Chen conceived the idea and designed the work. Binglin Yue, Haiyan Yang, Jian Wang, Wenxiu Ru, Jiyao Wu, Yongzheng Huang and Xianyong Lan integrated materials. Binglin Yue wrote the paper. Chuzhao Lei revised the manuscript critically. All authors have read and approved the final manuscript.

## Data Availability

Data sharing is not applicable to this article as no new data were created or analysed in this study.
